# *GSTM1*, *GSTT1* and *GSTP1* Ile105Val polymorphisms in outcomes of head and neck squamous cell carcinoma patients treated with cisplatin chemoradiation

**DOI:** 10.1038/s41598-019-45808-6

**Published:** 2019-06-27

**Authors:** Eder C. Pincinato, Ericka F. D. Costa, Leisa Lopes-Aguiar, Guilherme A. S. Nogueira, Tathiane R. P. Lima, Marília B. Visacri, Anna P. L. Costa, Gustavo J. Lourenço, Luciane Calonga, Fernanda V. Mariano, Albina M. A. M. Altemani, Cláudia Coutinho-Camillo, Carlos T. Chone, Celso D. Ramos, João M. C. Altemani, Patrícia Moriel, Carmen S. P. Lima

**Affiliations:** 1 Clinical Oncology Service, Department of Internal Medicine, School of Medical Sciences, University of Campinas, Campinas, São Paulo Brazil; 2 Health and Biological Science Center, Faculty of Pharmacy, Mackenzie Presbyterian University, São Paulo, São Paulo Brazil; 3 Faculty of Pharmaceutical Sciences, University of Campinas, Campinas, São Paulo Brazil; 4 Laboratory of Cancer Genetics, School of Medical Sciences, University of Campinas, Campinas, São Paulo Brazil; 5 Department of Ophthalmology and Otolaryngology, School of Medical Sciences, University of Campinas, University of Campinas, Campinas, São Paulo Brazil; 6 Department of Pathology, School of Medical Sciences, University of Campinas, São Paulo, Brazil; 7 A. C. Camargo Cancer Center, São Paulo, São Paulo Brazil; 8 Department of Radiology, School of Medical Sciences, University of Campinas, Campinas, São Paulo Brazil

**Keywords:** Cancer genetics, Cancer genetics, Head and neck cancer, Head and neck cancer

## Abstract

Cisplatin (CDDP) combined with radiotherapy (RT) is employed in head and neck squamous cell carcinoma (HNSCC) with variable toxicities and clinical response. Glutathione S-transferases (GSTs) participate in CDDP excretion from cells, and genes encoding GSTs, *GSTM1*, *GSTT1*and *GSTP1*, are polymorphic in humans. This prospective study aimed to evaluate the roles of *GSTM1*, *GSTT1*, and *GSTP1* Ile105Val polymorphisms in outcomes of HNSCC patients treated with CDDP chemoradiation. Ninety patients were genotyped by multiplex PCR. Urinary CDDP measurements were performed by HPLC. Treatment side effects and response were analysed by conventional criteria. Patients with *GSTT1* genes showed 7.23- and 5.37-fold higher likelihood of presenting vomiting and ototoxicity, lower glomerular filtration rate (GFR), and lower elimination of CDDP in urine relative to patients with deleted genes. Patients harbouring the *GSTP1* IleVal or ValVal genotypes showed 4.28-fold higher likelihood of presenting grade 2 or 3 vomiting and lower GFR with treatment than those harbouring the IleIle genotype. In multivariate Cox analysis, patients with the *GSTP1* 105ValVal genotype had 3.87 more chance of presenting disease progression than those with the IleIle or IleVal genotype (*p* < 0.01). Our findings provide preliminary evidence that inherited abnormalities in CDDP metabolism, related to *GSTT1* and *GSTP1* Ile105Val polymorphisms, alter outcomes of HNSCC patients treated with CDDP and RT.

## Introduction

Head and neck squamous cell carcinoma (HNSCC) is a common cancer affecting humans and is responsible for around 600,000 cases and 350,000 tumour-related deaths worldwide each year^1^.

Cisplatin (CDDP) is one of the first-line drugs against HNSCC and is usually administered with radiotherapy (RT)^2^. CDDP treatment leads to adducts with cellular DNA and triggers the release of free radicals, which induces cell death by apoptotic pathways^3^. RT induces lesions in cellular DNA via activation of photons and free radical generation, consequently leading to apoptosis of damaged cells. Therefore, the effects of RT are potentiated by CDDP^4^. Although the beneficial effects of CDDP are unequivocal, it is associated with numerous side effects involving the hematologic, gastrointestinal, renal, and auditory systems in HNSCC^5,6^. HNSCC patients treated with CDDP may also experience resistance and/or tumour recurrence within five years of follow-up^7^.

HNSCC patients with similar clinicopathological aspects are expected to present variable toxicity, tumour regression, and survival following treatment with CDDP-based chemoradiation^8^. Variability in patient responses can be caused by differences in genetic regulation of metabolic pathways.

Glutathione S-transferases (GSTs) participate in the excretion of CDDP from the cells^9^, and the genes encoding GSTs, namely, Mu 1 (*GSTM1*), Theta 1 (*GSTT1*), and Pi 1 (*GSTP1*), are polymorphic in humans. Null genotypes of *GSTM1* and *GSTT1* result in absence of encoded enzymes^10^, and the *GSTP1* Ile105Val polymorphism confers reduced enzymatic activity^11^. A few previous publications associated *GSTM1*, *GSTT1*, and/or *GSTP1* Ile105Val polymorphisms with variable toxicities in HNSCC patients treated with CDDP and RT^12–14^. In a previous study, tumour response to CDDP chemoradiation was observed in a HNSCC patient with distinct genotypes of *GSTT1* and *GSTP1* Ile105Val polymorphisms^12^; however, another study indicated that the *GSTM1*, *GSTT1*, and *GSTP1* genes did not alter response to CDDP^9^. Furthermore, the *GSTM1*, *GSTT1*, and/or *GSTP1* Ile105Val polymorphisms were associated with variable survival in HNSCC patients treated with CDDP chemoradiation^15^ or RT^16^. However, these genes showed no association with survival in radiation-treated HNSCC patients^17^.

Considering the limited number of studies that investigated these genes, we conducted this prospective study to determine whether the *GSTM1*, *GSTT1*, and *GSTP1* Ile105Val polymorphisms alter toxicity, response rate, and survival in HNSCC patients treated with CDDP and RT.

## Results

### Patients and laboratory characteristics

A total of 90 HNSCC patients with a median age of 56 years were enrolled in this study. The majority of the enrolled subjects were males, drinkers and smokers, and presented moderately differentiated and advanced tumours. Nearly two-thirds of the cases had tumours in the pharynx. All analysed cases tested negative for human papillomavirus (HPV) type 16. Most patients showed medium or high adherence to anti-emetics (Table 1).

**Table 1:** Clinicopathological and laboratory aspects of head and neck squamous cell carcinoma patients treated with cisplatin chemoradiation. (SD) standard deviation, (n) number of patients, (Crea) creatinine.

Variable	Median (range), mean ± SD, n (%)
**Median age (years)**	56 (27–74)
**Gender**	
Male	83 (92.2)
Female	7 (7.8)
**Drinking category**	
Absteiner	7 (7.8)
Light or moderate drinkers	19 (21.1)
Heavy or very heavy drinkers	64 (71.1)
**Smoking category**	
Non-smokers	2 (2.2)
Light or moderate smokers	9 (10.0)
Heavy smokers	79 (87.8)
**Tumor location**	
Oral cavity	12 (13.3)
Pharynx	55 (61.1)
Larynx	23 (25.6)
**Histological grade** ^*^	
Well or moderately	60 (82.2)
Poorly or undifferentiated	13 (17.8)
**Human papilomavirus 16** ^*^	
Positive	0 (0.0)
Negative	57 (100.0)
**Tumor stage** ^**^	
I or II	6 (6.7)
III or IV	84 (93.3)
**Adherence to anti-emetics** ^*^	
Medium or high adherence	86 (97.7)
Non-adherence	2 (2.3)
Median cumulative dose of intravenous CDDP (mg)	265 (100–616)
Mean cumulative urinary CDDP (μg CDDP/mg crea)***	237.0 ± 116.2
0–12 hs	71.75 ± 13.55
12–24 hs	8.61 ± 1.18
24–48 hs	6.71 ± 1.26

*The number of patients differed from the total quote in the study (n = 90), because no consistent information about histological grade, human papillomavirus type 16 status, and adherence to anti-emetics could be obtained in some cases. **According to the American Joint Committee on Cancer criteria. ***CDDP was measured in urine of 43 patients who had collected samples in all three cycles.

All patients were homogenously treated with RT at a total dose of 75 Gy and 80–100 mg/m^2^ of CDDP as initial dose as previously described (100 mg/m^2^ of CDDP was administered to patients with Karnofsky Performance Scale (KPS) 80–100% and without comorbidities, and patients with KPS 60–70% without comorbidities or KPS higher than 70% with comorbidities received CDDP at dose of 80 mg/m^2^). As recommended by the institutional protocol, 13 patients who presented adverse side effects of grades 3 or 4 received CDDP at lower dose (50–75 mg/m^2^) in further administrations or had suspension of CDDP. A total of 68 patients (75.5%) received three infusions of CDDP, while 22 patients (24.5%) received only two CDDP infusions because of hematologic or renal toxicities.

**Figure 1:**
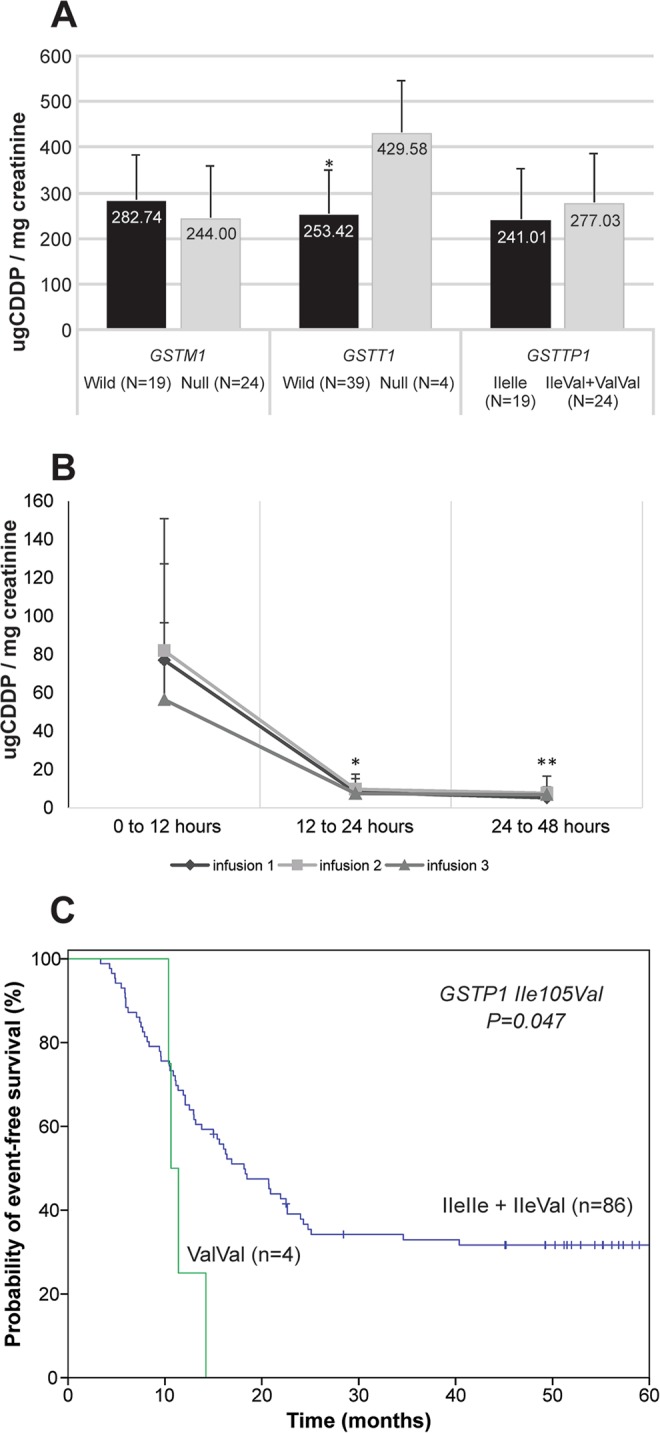
Urinary cisplatin (CDDP) and probability of event-free survival (EFS) of patients with head and neck squamous cell carcinoma treated with CDDP-chemoradiation. Average levels of CDDP after three CDDP infusion in 43 patients who collected urine in all three periods proposed in study, where *shows difference between CDDP excretion in 0–12 and 12–24 hours (*p* < 0.001), and **shows difference between CDDP excretion in 0–12 and 24–48 hours (*p* < 0.001) (**A**). Total CDDP urinary concentration in the same stratified by *GSTM1*, *GSTT1* and *GSTP1* Ile105Val genotypes, where *shows difference between patients with *GSTT1* null genotype and patients with *GSTT1* wild-type genotype (*p* = 0.04, power test: 83.4%) (**B**). Probabilities of EFS by Kaplan-Meier estimates in 90 patients stratified by genotypes of the *GSTP1* Ile105 Val polymorphism (**C**).

CDDP was measured in the urine samples of 43 patients after three CDDP infusions, and cumulative CCDP was found to be higher in urine samples collected in the first 12 h than in samples collected between 12 to 24 h (*p* = 0.001) and between 24 to 48 h (*p* = 0.001) (Table 1) (Fig. 1A). Similar urinary CDDP levels were observed in urine samples collected between 0 to 12 h (76.91 ± 50.26, 81.97 ± 68.74, and 56.39 ± 40.10 µg CDDP/mg creatinine; *p* = 0.06), 12 to 24 h (8.34 ± 7.02, 9.90 ± 7.82, and 7.59 ± 3.38 µg CDDP/mg creatinine, *p* = 0.10), and 24 to 48 h (5.35 ± 4.11, 7.83 ± 8.85, and 6.96 ± 4.10 µg CDDP/mg creatinine; *p* = 0.10) after the first, second, and third CDDP infusions, respectively.

Nearly four fifths and two thirds of available cases presented nausea and vomiting, respectively. The majority of patients showed hematologic toxicities; a quarter of the cases presented nephrotoxicity, and half of the patients showed ototoxicity (Table 2). Among the patients with available data on response rate (RR), 15 (20.5%) patients achieved complete response, 53 (72.6%) showed partial response (PR), and 5 (6.9%) showed stable disease (SD) status. Similar gastrointestinal and haematological toxicity (Table S1 Supplement), nephrotoxicity, ototoxicity, and response to therapy (Table S2 Supplement) were observed in patients stratified based on clinicopathological aspects and cumulative dose of intravenous CDDP.

**Table 2:** Toxicities to therapy in head and neck squamous cell carcinoma patients. (n) number of patients; (G) grade of toxicity.

Variable	Toxicity n (%)				
	G0	G1	G2	G3	G4
**Gastrointestinal toxicities**					
Nausea	15 (17.0)	22 (25.0)	40 (45.5)	11 (12.5)	0 (0.0)
Vomiting	38 (43.2)	21 (23.8)	19 (21.6)	10 (11.4)	0 (0.0)
**Hematologic toxicities**					
Anemia	2 (2.4)	35 (41.7)	32 (38.1)	15 (17.8)	0 (0.0)
Leukopenia	24 (28.6)	23 (27.4)	27 (32.1)	9 (10.7)	1 (1.2)
Thrombocytopenia	54 (64.3)	27 (32.1)	2 (2.4)	0 (0.0)	1 (1.2)
Nephrotoxicity	0 (0.0)	36 (52.2)	31 (44.9)	2 (2.9)	0 (0.0)
Ototoxicity	19 (27.1)	17 (24.3)	12 (17.2)	22 (31.4)	0 (0.0)

The total number of patients differed from the total enrolled in the study (n = 90), because consistent information about nausea and vomiting, hematologic exams, glomerular filtration rate or audiometry test after chemoradiotherapy was not obtained in some cases.

All cases were followed-up for a median period of 21 months (range: 3.0–74). The cumulative probabilities of event-free survival (EFS) and overall survival (OS) at 24 months follow-up were 35.0% and 40.0%, respectively. On the date of analysis (December 2017), 23 patients were alive, of which 6 had HNSCC and 17 did not have HNSCC. A total of 67 patients died, of which 59 mortalities were associated with tumour effects and eight were attributed to unrelated causes.

### Polymorphisms, toxicity, response, and CDDP excretion

The polymorphisms at the *GSTP1* Ile105Val locus in the analyzed participants were in Hardy-Weinberg equilibrium (HWE) (χ^2^ = 2.80, *p* = 0.09).

Patients harbouring the *GSTT1* genes were 7.23- and 5.37-fold more likely to present vomiting and ototoxicity of any grade (G1 to G3), respectively, relative to patients with the *GSTT1* null genotype. Patients with the *GSTP1* IleVal or ValVal genotype were 4.28-fold more likely to present vomiting (G2 or G3) than those carrying the IleIle genotype. Patients stratified based on nausea and nephrotoxicity symptoms showed similar frequencies of the *GSTM1*, *GSTT1*, and *GSTP1* Ile105Val polymorphisms (Table 3). In addition, patients harbouring different genotypes of the analysed polymorphisms showed no significant differences in haematological toxicity and response to therapy (Table S3 Supplement).

**Table 3:** Frequencies of *GSTM1*, *GSTT1* and *GSTP1* Ile105Val genotypes in head and neck squamous cell carcinoma patients stratified by gastrointestinal toxicity, nephrotoxicity and ototoxicity to chemoradiotherapy.

Variable	Nausea^*^ n (%)		Vomiting^*^ n (%)		Nephrotoxicity^*^ n (%)		Ototoxicity^*^ n (%)	
	G0	G1 to G3	G0	G1 to G3	G1	G2 to G5	G0	G1 to G4
***GSTM1***								
Present	8 (22.2)	28 (77.8)	13 (36.1)	23 (63.9)	15 (51.7)	14 (48.3)	10 (32.3)	21 (67.7)
Null	7 (13.5)	45 (86.5)	25 (48.1)	27 (51.9)	21 (52.5)	19 (47.5)	9 (23.1)	30 (76.9)
*p*-value	0.29		0.27		0.95		0.39	
OR (IC 95%)	0.54 (0.18–1.67)		1.64 (0.69–3.91)		1.03 (0.40–2.69)		0.63 (0.22–1.82)	
***GSTT1***								
Present	11 (15.1)	62 (84.9)	**26 (35.6)**	**47 (64.4)**	28 (50.0)	28 (50.0)	**12 (20.7)**	**46 (79.3)**
Null	4 (26.7)	11 (73.3)	**12 (80.0)**	**3 (20.0)**	8 (61.5)	5 (38.5)	**7 (58.3)**	**5 (41.7)**
*p*-value	0.28		0.004**		0.45		0.01***	
OR (IC 95%)	2.05 (0.55–7.61)		**7.23 (1.87–27.97)**		1.60 (0.47–5.50)		**5.37 (1.44–19.92)**	
***GSTP1***								
IleIle	8 (18.6)	35 (81.4)	22 (51.2)	21 (48.8)	16 (55.2)	13 (44.8)	9 (30.0)	21 (70.0)
IleVal or ValVal	7 (15.6)	38 (84.4)	16 (35.6)	29 (64.4)	20 (50.0)	20 (50.0)	10 (25.0)	30 (75.0)
*p*-value	0.70		0.14		0.67		0.64	
OR (IC 95%)	1.24 (0.41–3.79)		1.90 (0.81–4.46)		1.23 (0.47–3.21)		1.29 (0.45–3.71)	
IleIle or IleVal	14 (16.7)	70 (83.3)	37 (44.0)	47 (56.0)	33 (50.8)	32 (49.2)	18 (27.3)	48 (72.7)
ValVal	1 (25.0)	3 (75.0)	1 (25.0)	3 (75.0)	3 (75.0)	1 (25.0)	1 (25.0)	3 (5.0)
*p*-value	0.67		0.46		0.37		0.92	
OR (IC 95%)	0.60 (0.06–6.20)		2.36 (0.24–23.65)		0.34 (0.03–3.48)		1.12 (0.11–11.53)	

(n) number of patients; (G) grade of toxicity; (OR) odds ratio; (CI) confidence interval. (*) The total number of patients differed from the total (n = 90), because consistent information about response rate, nausea, vomiting or audiometry test was not obtained in some cases. Vomiting G0 or G1 were seen in 35 out of 43 (81.4%) and 24 out of 45 (53,3%) patients with IleIle and IleVal or ValVal genotype, respectively, and vomiting G2 or G3 were seen in 8 out 43 (18.6%) and 21 out of 45 (46,7%) patients with IleIle and IleVal or ValVal genotype, respectively (***p*** = 0.004****); patients with IleVal or ValVal genotype had 4.28 (IC 95%: 1.58–11.60) more chance of presenting vomiting G2 or G3 than patients with the IleIle genotype. **power test: 96.5% and *p*bootstrap = 0.002, ***power test: 82.8% and *p*bootstrap = 0.006, and ****power test: 88.9% and *p*bootstrap = 0.002. Significant differences between groups are presented in bold letters.

Reduction in glomerular filtration rate (GFR) after chemoradiation was found to be more pronounced in patients harbouring the *GSTT1* genes and in patients with *GSTP1* IleVal or ValVal genotype relative to patients with the *GSTT1* null genotype and IleIle genotype, respectively (Table 4). The mean cumulative urinary CDDP level (in the three cycles) was higher in patients with the *GSTT1* null genotype than in those carrying the *GSTT1* gene (429.58 ± 116.24 *vs*. 253.42 ± 95.20 g CDDP/mg creatinine, *p* = 0.04; power test = 83.4%) (Fig. 1B). A unique patient with combined *GSTM1* null, *GSTT1* null, and *GSTP1* IleIle genotypes showed the highest reported total CDDP elimination rate (545.42 µg CDDP/mg creatinine).

**Table 4:** Frequencies of *GSTM1*, *GSTT1* and *GSTP1* Ile105Val genotypes in head and neck squamous cell carcinoma patients stratified by glomerular filtration rate.

Variable	n	^51^Cr-EDTA GFR (mL/min/1.73 m²)	
		Before treatment Mean ± SD	After treatment Mean ± SD
GSTM1*			
Present	29	82.05 ± 20.50	64.01 ± 19.65
Null	40	84.50 ± 19.89	64.34 ± 21.97
p-value		0.45	
GSTT1*			
Present	56	**84.38 ± 19.96**	**62.87 ± 20.72**
Null	13	**79.56 ± 20.68**	**69.94 ± 21.40**
*p*-value		**0.04**	
***GSTP1*** Ile105Val*			
IleIle	29	**80.87 ± 21.73**	**66.97 ± 24.96**
IleVal or ValVal	40	**85.35 ± 18.76**	**62.19 ± 17.40**
*p*-value		**0.03**	
IleIle or IleVal	65	82.65 ± 19.86	63.23 ± 20.52
ValVal	4	96.88 ± 20.78	79.98 ± 23.27
*p*-value		0.55	

(n) number of patients; (^51^Cr-EDTA GFR) glomerular filtration rate measured with EDTA labelled with chrome^37^; (SD) standard deviation. *The total number of patients differed from the total enrolled in study (n = 90), because it was not possible to obtain consistent information in some cases. Significant differences between groups are presented in bold letters.

### Polymorphisms and survival analysis

At 24 months of follow-up, patients with advanced tumours (26.3% *vs*. 83.3%, *p* = 0.02) and patients with the *GSTP1* ValVal genotype (0.0% *vs*. 31.7%, *p* = 0.047) had shorter event-free survival (EFS) than those with localised tumours and those harbouring the *GSTP1* IleVal or ValVal genotype (Fig. 1C). Only patients with advanced tumours showed shorter OS (83.3% *vs*. 21.7%, *p* = 0.009) (Kaplan-Meier estimates). In univariate Cox analysis, patients with advanced tumours showed 7.50- and 8.88-fold higher likelihood of disease progression and mortality than those with localised tumours, respectively. Results of multivariate Cox analysis showed that patients with advanced tumours and patients harbouring the *GSTP1* 105ValVal genotype had 8.90- and 3.87-fold higher likelihood of presenting disease progression than those with localised tumours and harbouring the *GSTP1* IleIle or Ile Val genotype, respectively. The significance of survival differences were confirmed by the bootstrapping method (Table 5).

**Table 5:** Association of clinicopathological characteristics and *GSTM1*, *GSTT1* and *GSTP1* Ile105Val genotypes with survival of head and neck squamous cell carcinoma patients treated with chemoradiotherapy in univariate and multivariate Cox analyses.

Variable	Event-free survival				HR (95% CI)	Overall survival		
	n with event/n total	Univariate analysis		Multivatiate analysis		n with event/n total	Univariate analysis	
		*p*-value	HR (95% CI)	*p*-value			*p*-value	HR (95% CI)
**Age (years)**								
≤56	26/43					32/43		
>56	31/47	0.26	1.33 (0.81–2.19)	NA	NA	35/47	0.76	1.08 (0.67–1.74)
**Gender**								
Male	57/83					63/83		
Female	6/7	0.30	1.25 (0.82–1.91)	NA	NA	4/7	0.61	1.30 (0.47–3.57)
**Tobacco consumption**								
Smokers	61/88					1/2		
Non-smokers	2/2	0.12	1.75 (0.86–3.56)	NA	NA	66/88	0.68	1.51 (0.21–10.91)
**Alcohol consumption**								
Drinkers	60/83					63/83		
Absteiners	3/7	0.28	1.91 (0.60–6.10)	NA	NA	4/7	0,27	1.78 (0.64–4.90)
**Tumor location**								
Oral cavity ororopharynx	38/51					37/51		
Hypopharynx or larynx	25/39	0.44	1.22 (0.74–2.03)	NA	NA	26/39	0.52	1.17 (0.72–1.91)
**Histological grade**								
Well or moderately	42/60					40/60		
Poorly or undifferentiated	9/13	0.41	1.36 (0.66–2.79)	NA	NA	10/13	0.51	1.26 (0.63–2.51)
**Tumor stage**								
I or II	1/6					1/6		
III or IV	62/84	**0.04**	**7.50 (1.04–54.20)** ^*^	**0.03**	**8.90 (1.22–64.99)** ^**^	62/84	**0.03**	**8.88 (1.23–64.12)** ^***^
***GSTM1***								
Present	24/38					26/38		
Null	39/52	0.18	1.41 (0.85–2.36)	NA	NA	37/52	0.16	1.43 (0.87–2.34)
***GSTT1***								
Present	53/75					53/75		
Null	10/15	0.54	1.24 (0.63–2.43)	NA	NA	10/15	0.38	1.35 (0.69–2.65)
***GSTP1*** **Ile105Val**								
IleIle	32/43					31/43		
IleVal or ValVal	31/47	0.20	0.73 (0.44–1.19)	NA	NA	32/47	0.16	1.41 (0.87–2.28)
IleIle or IleVal	59/86					60/86		
ValVal	4/4	0.052	2.75 (0.97–7.80)	**0.01**	**3.87 (1.34–11.17)** ^****^	3/4	0.92	1.06 (0.33–3.38)

(n) number of patients; (HR) hazard ratio; (CI) confidence interval; (NA) Not aplicable. Multivariate analysis was adjusted by tumour stage and *GSTP1* Ile105Val genotypes. ^*^pbootstrap = 0.03; ^**^pbootstrap = 0.01, ^***^pbootstrap = 0.02, ^****^pbootstrap = 0.001. Significant differences between groups are presented in bold letters.

## Discussion

Our findings on the clinicopathological aspects of HNSSC patients, toxicity and RR to chemoradiation, and survival were similar to those described in previous studies^18,19^. A history of tobacco and alcohol consumption but the low prevalence of HPV infection indicate that history of tobacco and alcohol consumption were in fact the most important factors influencing tumour development in the analysed patients.

The presence of *GSTT1* and the *GSTP1* IleVal or ValVal genotype were found to be associated with a higher likelihood of presenting vomiting with chemoradiation, as previously suggested^20^. In a previous publication, we analysed almost the same patients enrolled in the current study (n = 88) and focused on the roles of *GSTM1*, *GSTT1*, *GSTP1*, and other genetic polymorphisms in genes involved in the repair of DNA damage induced by CDDP and apoptosis of cells with CDDP-induced lesions, only in severity of vomiting presented by HNSCC patients treated with CDDP chemoradiation^14^. Our previous findings indicated that the *GSTP1* IleVal or ValVal genotype, but not the presence of *GSTT1*, was associated with vomiting; in the current study, vomiting was strongly associated with the presence of both *GSTT1* and the *GSTP1* IleVal or ValVal genotype. The contrasting results between the two studies can be attributed to the differences in patient stratification based on the severity of vomiting grade. In the previous publication, patients were stratified based on grade 0 or 1 *vs*. grade 2 or 3, whereas patients in the present study (n = 90) were stratified based on grade 0 *vs*. grades 1 to 3.

*GSTT1* null patients were expected to show more severe vomiting than patients harbouring *GSTT1*; however, opposite results were obtained in the present study. Nutrient deficiency in patients with malignancies was postulated to lead to a lack of GSH, thereby causing impaired detoxification of free radicals of CDDP by GSTT1 and more severe toxicity^13^. In fact, reduction in body mass was found to be more predominantly observed in HNSCC patients receiving CDDP and RT treatment^21^, which could have modified the effects of the *GSTT1* gene.

CDDP is well known to cause vomiting by inducing DNA damage in epithelial enterocromaffin cells of the intestine, thereby leading to serotonin release and stimulation of the chemoreceptor trigger zone and vomiting centre^22^. On the other hand, in addition to its role in CDDP detoxification, GSTP1 regulates JNK signalling pathways by forming a complex with c‐Jun‐JNK, which inactivates JNK signalling and inhibits apoptosis^23^. Thus, increased apoptosis of CDDP-damaged enterochromaffin cells of the intestines, which is associated with the less active “Val” allele of the *GSTP1* Ile105Val polymorphism, may have influenced the severity of vomiting in the HNSCC patients.

In addition, the presence of *GSTT1* was associated with ototoxicity in the patients. Oldenburg *et al*.^24^ demonstrated that the *GSTM1* null genotype conferred protection against hearing impairment in testicular cancer patients treated with CDDP. Talach *et al*.^13^ reported that early treatment with CDDP induced ototoxicity in adults with various tumours types, including HNSCC, and two copies of the *GSTT1* gene. The *GSTP1* “Val” allele was associated with ototoxicity in patients with medulloblastoma who were treated with CDDP-based therapy^25^.

CDDP induces ototoxicity through the generation of free radicals and subsequently activates cell death pathways, such as the c-Jun N terminal kinase (JNK) and p38 mitogen activated protein kinase (MAPK) pathways, which in turn induce hair cell apoptosis and hearing loss^26^. GSTs represent the major enzyme family involved in the excretion of CDDP, which protects cells from the deleterious effects of free radicals^9^. Again, we expected more severe ototoxicity in *GSTT1* null patients than in patients harbouring *GSTT1*; however, contrasting results were obtained in the present study. Talach *et al*.^13^ observed that patients with testicular tumours tended to have increased ototoxicity and presence of *GSTT1*. They postulated that nutrient deficiency with lack of GSH production and impaired detoxification of free radicals of CDDP in the sensory inner ear cells consequently increased ototoxicity. The same mechanisms are likely to occur in HNSCC patients in the present study.

Our current findings indicated that the *GSTM1*, *GSTT1*, and *GSTP1* Ile105Val polymorphisms did not alter myelotoxicity and RR to CDDP and RT. Cabelguenne *et al*.^9^ found no associations of the *GSTM1*, *GSTT1*, and *GSTP1* Ile105Val polymorphisms with RR in HNSCC patients treated with low doses of CDDP (25 mg/m²) and 5-FU. We observed pronounced myelotoxicity and a durable complete response in one HNSCC patient treated with both CDDP (100 mg/m²) and RT, which was attributed to inherited deficiency in CDDP detoxification (*GSTT1* null genotype), deficiency in repair of CDDP cellular damage (*MSH3* 1045ThrThr), and increased apoptosis induction during CDDP cellular damage (*GSTP1* 105IleIle)^12^.

Patients harbouring the *GSTT1* and *GSTP1* IleVal or ValVal genotype showed more pronounced reduction in ^51^Cr-EDTA GFR after chemoradiation than those with the remaining genotypes. Khrunin *et al*.^27^ observed that patients with epithelial ovarian cancer and harbouring the *GSTT1* null genotype who were treated with CDDP (100 mg/m^2^) plus cyclophosphamide were 3.31-fold more likely to develop nephrotoxicity.

Given its low molecular weight, CDDP is conjugated with reduced glutathione (GSH) in the liver. CDDP-GSH is freely filtered in the glomerulus and is completely recovered in the urine. CDDP-GSH reaches high concentrations in the proximal tubular cells of the internal renal cortex and the outer medullary layer. The mechanism of CDDP-induced tubular damage is complex and involves several mechanisms, such as CDDP accumulation-mediated membrane transport, conversion to nephrotoxin, DNA damage, mitochondrial dysfunction, oxidative stress, inflammatory response, activation of transducers and intracellular messengers, and activation of apoptotic pathways^28^.

To our knowledge, the roles of GSTT1 and GSTP1 in renal damage by CDDP remain unknown. Thus, the high CDDP-GSH concentrations in proximal tubule cells, associated with the *GSTT1* genes, are likely to influence the severity of renal dysfunction in our patients. Furthermore, patients harbouring the *GSTP1* IleVal or ValVal genotype showed more pronounced renal dysfunction. These results are unexpected because the protein encoded by the “Val” allele is known to be less efficient in CDDP-GSH production than that encoded by the “Ile” allele^11^. Again, this association could be attributed to effects of the protein encoded by the “Val” allele, which induces apoptosis in proximal tubule cells^23^.

Nearly 90% of CDDP excretion in our patients was measured within the first 24 h after infusion. Patients with the different *GSTT1* genotypes showed variable CDDP levels, suggesting that gene polymorphisms influence the kinetics of CDDP elimination. Notably, pharmacokinetic studies usually analyse plasma samples obtained from patients receiving therapy. However, in the present study, sequential blood plasma collection cannot be obtained from outpatients. Therefore, we determined CDDP levels in urine for this pharmacokinetic analysis. The HPLC method used was well established^29^ and considered essential to pharmacokinetics and pharmacodynamics studies^30^. Lanjwani *et al*.^29^ postulated a correlation between urine and plasma CCDP levels and that both parameters could be used to pharmacokinetics studies of the drug. To the best of our knowledge, our study is the first to analyse the effects of *GST* polymorphisms on CDDP pharmacokinetics in HNSCC patients. Results of a unique study conducted by Joerger *et al*.^31^ showed no associations between *GSTM1* and *GSTP1* A313G (Ile105Val) genotypes with CDDP pharmacokinetics in advanced non-small-cell lung cancer patients.

Surprisingly, patients with the *GSTT1* null genotype showed higher cumulative urinary CDDP levels compared to patients with the *GSTT1* genotype. In this case, the deficiency in an individual GST isoenzyme could be compensated for by other isoforms^32^. Consistent with the above hypothesis, the only patient with *GSTM1* null, *GSTT1* null, and *GSTP1* IleIle genotypes showed the highest total CDDP elimination rate.

Finally, our findings indicated that patients carrying the *GSTP1* Ile105Val polymorphism had altered EFS. There were no associations between the *GSTM1* and *GSTT1* genotypes and survival in early-stage radiation-treated HNSCC patients^17^. In addition, disease-specific survival was observed in patients with SCC of oral cavity and harbouring *GSTM1* who were treated with radiotherapy^16^. Shorter survival rates were observed in *GSTM1* null patients with SCC of oral cavity who were treated with CDDP-based chemotherapy^15^. To the best of our knowledge, no published studies have focused on the role of the *GSTP1* Ile105Val polymorphism in survival of HNSCC patients treated with CDDP and RT. The *GSTP1* 105IleIle genotype was previously associated with higher PFS in epithelial ovarian cancer patients treated with CDDP (100 mg/m²) and cyclophosphamide^27^ and oesophageal cancer patients treated with CDDP and 5-FU/paclitaxel^33^. The observed association between *GSTP1* ValVal genotype and shorter EFS in the current study was based on a small sample size and cannot be explained by direct drug detoxification by GSTP1, which will lead to higher survival. Free radicals produced by CDDP that are not detoxified due to the lack of GSTP1 are likely to induce new mutations in residual tumour cells, thereby facilitating survival and proliferation of the tumour cells^34^.

In summary, our study is the first to provide preliminary evidence that inherited *GSTT1* and *GSTP1* Ile105Val polymorphisms can alter gastrointestinal status, nephrotoxicity, ototoxicity, pharmacokinetics, and survival in HNSCC patients treated with CDDP chemoradiation. In this context, GST genotypes can be used as instruments for assessing toxicity of CDDP-based therapies by selecting HNSCC patients for the use of specific anti-emetics and protective renal and auditory system agents, thereby saving patients from the side effects of chemotherapy^14,35,36^ and reducing treatment costs^37^. However, we are aware that the number of patients enrolled in study was not large, and that further larger studies and functional analyses of relevant polymorphisms are required to confirm the roles of these *GST*s in disease.

## Material and Methods

### Subjects and clinical variables

In this prospective study, HNSCC patients diagnosed at the Clinical Oncology Service of the General Hospital of University of Campinas between June 2011 and February 2014 were selected to undergo CCDP chemoradiation as definitive treatment due to locoregional unresectable tumour, refusal of surgery related to expected functional, or anatomic sequels or an organ preservation protocol. The following exclusion criteria were considered: (1) refusal to participate in the study; (2) low KPS score; (3) renal dysfunction; (4) previous hearing dysfunction; and/or (5) other therapeutic protocol. The present study was conducted according to the Declaration of Helsinki and was approved by the Ethics Committees of the University of Campinas (n° 274/2011; CAAE: 0218.0.146.000-11). All patients provided written informed consent after they were informed about the study and any associated risks.

Data relating to age, gender, tobacco and alcohol consumption, hematologic and biochemistry exams, tumour location, histological grade, and stage were obtained using specific questionnaires or patient charts. Subjects were classified according to smoking^38^ and drinking habits^39^ as previously reported. Tumours were diagnosed based on standard criteria^40^ and staged based on criteria specified by the American Joint Committee on Cancer^41^. HPV type 16 was investigated in tumour fragments by immunohistochemistry and *in situ* hybridization as previously reported^42^.

Toxicities (nausea, vomiting, hematologic, nephrotoxicity, and ototoxicity) were assessed using data on adverse effects, hematologic exams, GFR measured with EDTA labelled with chrome^37^ (^51^Cr-EDTA GFR), and audiometric tests performed before and after chemoradiotherapy. Toxicities were evaluated according to the National Cancer Institute based on common terminology criteria for adverse events version 4.0 (CTCAE)^43^. For each patient, the worst grade for each toxicity was included in analysis.

Patients were homogeneously treated with CDDP chemoradiation according to the institutional protocol^42^. RR to treatment was assessed using the Response Evaluation Criteria in Solid Tumors (RECIST) guidelines version 1.1^44^. Surgical tumour resection was indicated to patients with good clinical condition and partial response or tumour relapse.

On each day of CDDP infusion, patients received hydration (3 L of 0.9% saline solution), 125 mL of 20% mannitol as diuretic, 20 mL of 19.1% potassium chloride and 10 mL of magnesium sulphate as electrolytes, and 20 mg of dexamethasone plus 24 mg of ondansetron as prophylaxis to acute emesis. Patients were orally administered with 8 mg of dexamethasone (every 12 h) and 10 mg of metoclopramide (every 6 h) for three additional days^42^. Anti-emetic adherence was classified as previously described^42^.

Patient follow-up was performed in three-month intervals. The end of the follow-up period was September 2018.

### DNA extraction and genotyping

Genomic DNA of all subjects was isolated from 5 mL of peripheral blood sample following the proteinase K and lithium chloride method^45^.

*GSTM1* and *GSTT1* genotypes were amplified from genomic DNA by the multiplex-polymerase chain reaction (multiplex-PCR)^46^. *GSTP1* Ile105Val genotypes were identified by PCR and enzymatic digestion^47^. Positive and negative controls were used in all genotyping reactions. The amount of 15% of genotype determinations was carried out twice in independent experiments and showed 100% of concordance.

### Urinary CCDP excretion kinetics

Urine was collected from patients by voluntary urination during three different collection periods: 0–12 h, 12–24 h, and 24–48 h after each CDDP infusion. Samples were stored at −80 °C until analysis^48^.

CDDP derivatization and extraction from patient urine and a sample of standard urine (without CDDP) were conducted as previously described^49^. Nickel chloride and chloroform were obtained from Merck, Darmstadt, Germany, and diethyldithiocarbamate was obtained from Sigma-Aldrich, India. Samples were analysed using a high-performance liquid chromatography (HPLC) Separation Module system with dual absorbance detector (UV-visible detector, wavelength 254 nm) (Waters 2487, Milford, MA, USA) and a Hypersil ODS C18 column (150 mm × 4 mm with a particle size of 4 μm (Thermo, Waltham, MA, USA). The HPLC conditions and the mobile phase were set as described by Lopez-Flores *et al*.^50^. Sample measurement and standard curves were performed in duplicate. Urinary CDDP was normalized with urinary creatinine and measured using akinetic kit (Creatinine Laborclin kit, Parana, Brazil). The final concentration of urinary CDDP was calculated as the sum of all measurements obtained after each CDDP administration.

### Statistical analysis

The HWE was tested with chi-square (χ^2^) statistic for the goodness-to-fit. Differences between groups were analysed by χ^2^ or Fisher’s exact test. A logistic regression model was generated to obtain odds ratios (OR) values to verify associations between genotypes, nausea, vomiting, nephrotoxicity, ototoxicity, haematological toxicity, and RR. Comparisons between GFR before and after CCDP treatment were evaluated by two-way ANOVA. Student’s *t*-test was performed to determine associations between average CDDP urinary elimination rate in each collection period (0–12, 12–24, and 24–48 h) and to determine significant associations of the total CDDP elimination rates with genotypes. The statistical power of a test was calculated using the Researcher’s Toolkit^51^.

EFS and OS were calculated for each participant from the date of diagnosis until the date of tumour progression, tumour relapse, death attributed to tumour effects, or last follow-up and from the date of diagnosis until the date of death by any cause or last follow-up contact, respectively. Kaplan-Meier method was used to plot EFS and OS curves, and log-rank test was conducted to determine significant differences between curves. Multivariate Cox regression was used to estimate hazard ratios (HRs) adjusted for potential possible discrepancies in clinical aspects (*p* ≤ 0.10 in univariate Cox regression). Bootstrapping (n = 1,000) based on repeatedly random sampling was applied to ensure the stability of the model by applying the bias-corrected and accelerated method.

*P*-values were two-sided and considered statistically significant at *p* < 0.05. Statistical analyses were conducted using SPSS 21.0 software (IBM Corporation, Armonk, NY, USA).

## Supplementary information

Supplement

## Data Availability

The authors declare that all data of this study are available from the corresponding author upon reasonable request.
