# Supplementary material for: GSTM1, GSTT1 and GSTP1 Ile105Val polymorphisms in outcomes of head and neck squamous cell carcinoma patients treated with cisplatin chemoradiation
Source: Sci Rep. 2019 Jun 27;9:9312. doi: 10.1038/s41598-019-45808-6 (PMC6597539; doi:10.1038/s41598-019-45808-6)
Supplement: Supplementary file 1 — Supplement [file 41598_2019_45808_MOESM1_ESM.docx]

***GSTM1*, *GSTT1* and *GSTP1* Ile105Val polymorphisms in outcomes of head and neck squamous cell carcinoma patients treated with cisplatin chemoradiation**

Eder C Pincinato^1,2^, Ericka F D Costa^1^, Leisa Lopes-Aguiar^1^, Guilherme A S Nogueira^1^, Tathiane R P Lima^1^, Marília B Visacri^3^, Anna P L Costa^3^, Gustavo J Lourenço^4^, Luciane Calonga^5^, Fernanda V Mariano^6^, Albina M A M Altemani^6^, Cláudia Coutinho-Camillo^7^, Carlos T Chone^5^, Celso D Ramos^8^, João M C Altemani^8^, Patrícia Moriel^3^ & Carmen S P Lima^1,*^

**^1^** Clinical Oncology Service, Department of Internal Medicine, School of Medical Sciences, University of Campinas, Campinas, São Paulo, Brazil

**^2^** Health and Biological Science Center, Faculty of Pharmacy, Mackenzie Presbyterian University, São Paulo, São Paulo, Brazil

**^3^** Faculty of Pharmaceutical Sciences, University of Campinas, Campinas, São Paulo, Brazil

**^4^** Laboratory of Cancer Genetics, School of Medical Sciences, University of Campinas, Campinas, São Paulo, Brazil

**^5^** Department of Ophthalmology and Otolaryngology, School of Medical Sciences, University of Campinas, University of Campinas, Campinas, São Paulo, Brazil

**^6^** Department of Pathology, School of Medical Sciences, University of Campinas, São Paulo, Brazil

**^7^** A. C. Camargo Cancer Center. São Paulo, São Paulo, Brazil

**^8^** Department of Radiology, School of Medical Sciences, University of Campinas, Campinas, São Paulo, Brazil

**Corresponding author***

**Carmen S P Lima, MD, PhD**

Clinical Oncology Service, Department of Internal Medicine

Faculty of Medical Sciences, University of Campinas

Cidade Universitária “Zeferino Vaz”

Campinas, São Paulo, Brazil. CEP: 13083-887

Phone and fax simile: +55 19 3521 9120

E-mail: [carmenl@fcm.unicamp.br](mailto:carmenl@fcm.unicamp.br)

| **Aspects** | **Nausea* median (range) or n (%)** | | | **Vomiting* median (range) or n (%)** | | |
| --- | --- | --- | --- | --- | --- | --- |
|  | **G0** | **G1 to G3** | ***p*-value** | **G0** | **G1 to G3** | ***p*-value** |
| **Age** | 54 (30-72) | 56 (27-74) | 0.37 | 56 (30-74) | 54 (27-69) | 0.25 |
| **Gender** |  |  |  |  |  |  |
| Male | 14 (93.3) | 67 (91.8) | 0.84 | 35 (92.1) | 46 (92.0) | 0.99 |
| Female | 1 (6.7) | 6 (8.2) |  | 3 (7.9) | 4 (8.0) |  |
| **Drinking category** |  |  |  |  |  |  |
| Absteiner | 1 (6.7) | 6 (8.2) | 0.84 | 3 (7.9) | 4 (8.0) | 0.98 |
| Drinkers | 14 (93.3) | 67 (91.8) |  | 35 (92.1) | 46 (92.0) |  |
| **Smoking category** |  |  |  |  |  |  |
| Non-smokers | 1 (6.7) | 1 (1.4) | 0.26 | 1 (2.6) | 1 (2.0) | 0.84 |
| Smokers | 14 (93.3) | 72 (98.6) |  | 37 (97.4) | 49 (98.0) |  |
| **Tumor location** |  |  |  |  |  |  |
| Oral cavity | 2 (13.3) | 10 (13.7) | 0.83 | 5 (13.2) | 7 (14.0) | 0.99 |
| Pharynx | 10 (66.7) | 43 (58.9) |  | 23 (60.5) | 30 (60.0) |  |
| Larynx | 3 (20.0) | 20 (27.4) |  | 10 (26.3) | 13 (26.0) |  |
| **Histological grade** |  |  |  |  |  |  |
| Well or moderately | 9 (69.2) | 50 (84.7) | 0.99 | 28 (84.8) | 31 (79.5) | 0.56 |
| Poorly or undifferentiated | 4 (30.8) | 9 (15.2) |  | 5 (15.2) | 8 (20.5) |  |
| **Tumor stage** |  |  |  |  |  |  |
| I or II | 0 (0.0) | 6 (8.2) | 0.99 | 3 (7.9) | 3 (6.0) | 0.73 |
| III or IV | 15 (100.0) | 67 (91.8) |  | 35 (92.1) | 47 (94.0) |  |
| **Adherence to anti-emetics** |  |  |  |  |  |  |
| Medium or high | 15 (100.0) | 71(97.3) | 0.99 | 38 (100.0) | 48 (96.0) | 1.00 |
| Non-adherence | 0 (0.0) | 2 (2.7) |  | 0 (0.0) | 2 (4.0) |  |
| **Cumulative dose of CDDP (mg/m²)** | 243 (100-300) | 250 (140-300) | 0.66 | 241 (100-300) | 254 (140-300) | 0.20 |

**Table S1.** Clinicopathological aspects and cumulative dose of cisplatin in gastrointestinal toxicity in head and neck squamous cell carcinoma patients treated with chemoradiation. (n) number of patients; (G) grade of toxicity; (CDDP) cisplatin. *The number of patients differed from the total (n = 90), because consistent information about gastrointestinal toxicity was not obtained in some cases.

| **Aspects** | **Nephrotoxicity* median (range) or n (%)** | | | **Ototoxicity* median (range) or n (%)** | | | **Response rate* median (range) or n (%)** | | |
| --- | --- | --- | --- | --- | --- | --- | --- | --- | --- |
|  | **G1** | **G2 to G5** | ***p*-value** | **G0** | **G1 to G4** | ***p*-value** | **CR or PR** | **SD** | ***p*-value** |
| **Age** | 54 (27-66) | 56 (37-73) | 0.33 | 58 (30-73) | 54 (27-69) | 0.08 | 55 (30-73) | 51 (37-62) | 0.30 |
| **Gender** |  |  |  |  |  |  |  |  |  |
| Male | 31 (86.1) | 32 (97.0) | 0.14 | 17 (89.5) | 47 (92.2) | 0.72 | 64 (94.1) | 4 (80.0) | 0.26 |
| Female | 5 (13.9) | 1 (3.0) |  | 2 (10.5) | 4 (7.8) |  | 4 (5.9) | 1 (20.0) |  |
| **Drinking category** |  |  |  |  |  |  |  |  |  |
| Absteiner | 4 (11.1) | 1 (3.0) | 0.23 | 2 (10.5) | 3 (5.9) | 0.51 | 3 (4.4) | 1 (20.0) | 0.18 |
| Drinkers | 32 (88.9) | 32 (97.0) |  | 17 (89.5) | 48 (94.1) |  | 65 (95.6) | 4 (80.0) |  |
| **Smoking category** |  |  |  |  |  |  |  |  |  |
| Non-smokers | 1 (2.8) | 0 (0.0) | 1.00 | 0 (0.0) | 1 (2.0) | 1.00 | 0 (0.0) | 0 (0.0) | NE |
| Smokers | 35 (97.2) | 33 (100.0) |  | 19 (100.0) | 50 (98.0) |  | 68 (100.0) | 5 (100.0) |  |
| **Tumor location** |  |  |  |  |  |  |  |  |  |
| Oral cavity | 5 (13.9) | 3 (9.1) | 0.63 | 2 (10.5) | 8 (15.7) | 0.55 | 7 (10.3) | 1 (20.0) | 0.79 |
| Pharynx | 20 (55.6) | 22 (66.7) |  | 11 (57.9) | 33 (64.7) |  | 43 (63.2) | 3 (60.0) |  |
| Larynx | 11 (30.6) | 8 (24.2) |  | 6 (31.6) | 10 (19.6) |  | 18 (26.5) | 1 (20.0) |  |
| **Histological grade** |  |  |  |  |  |  |  |  |  |
| Well or moderately | 25 (89.3) | 21(77.8) | 0.26 | 14 (77.8) | 33 (86.8) | 0.39 | 43 (81.1) | 5 (100.0) | 0.99 |
| Poorly or undifferentiated | 3 (10.7) | 6 (22.2) |  | 4 (22.2) | 5 (13.2) |  | 10 (18.9) | 0 (0.0) |  |
| **Tumor stage** |  |  |  |  |  |  |  |  |  |
| I or II | 3 (8.3) | 1 (3.0) | 0.37 | 0 (0.0) | 4 (7.8) | 0.99 | 3 (4.4) | 0 (0.0) | 0.99 |
| III or IV | 33 (91.7) | 32 (97.0) |  | 19 (100.0) | 47 (92.2) |  | 65 (95.6) | 5 (100.0) |  |
| **Adherence to anti-emetics** |  |  |  |  |  |  |  |  |  |
| Medium or high | 36 (100.0) | 33 (100.0) | 0.72 | 19 (100.0) | 50 (98.0) | 0.39 | 67 (98.5) | 5 (100.0) | 1.00 |
| Non-adherence | 0 (0.0) | 0 (0.0) |  | 0 (0.0) | 1 (2.0) |  | 1 (1.5) | 0 (0.0) |  |
| **CDDP dose (mg/m²)** | 249 (160-300) | 249 (100-300) | 0.99 | 233 (100-300) | 256 (140-300) | 0.07 | 249 (100-300) | 252 (200-300) | 0.88 |

**Table S2.** Clinicopathological aspects and cumulative dose of cisplatin in nephrotoxicity, ototoxicity and response to chemoradiation in head and neck squamous cell carcinoma patients. (n) number of patients; (G) grade of toxicity; (CR) complete response; (PR) partial response; (SD) stable disease; (SD) standard deviation; (NE) not evaluated because of the low number of patients; (CDDP) cisplatin. ^*^The total number of patients differed from the total (n = 90), because consistent information about nephrotoxicity, ototoxicity and response rate was not obtained in some cases.

| **Variable** | **Anemia* n (%)** | | **Leukopenia* n (%)** | | **Thrombocytopenia* n (%)** | | **Response rate* n (%)** | |
| --- | --- | --- | --- | --- | --- | --- | --- | --- |
|  | **G0** | **G1 to G3** | **G0** | **G1 to G4** | **G0** | **G1 to G4** | **RC or RP** | **SD** |
| ***GSTM1*** |  |  |  |  |  |  |  |  |
| Present | 0 (0.0) | 35 (100.0) | 9 (25.7) | 26 (74.3) | 14 (66.7) | 7 (33.3) | 30 (96.8) | 1 (3.2) |
| Null | 2 (4.1) | 47 (95.9) | 15 (30.6) | 34 (69.4) | 12 (42.9) | 16 (57.1) | 38 (90.5) | 4 (9.5) |
| *p*-value | 1.00 | | 0.62 | | 0.10 | | 0.31 | |
| OR (IC 95%) | NE | | 0.78 (0.30-2.07) | | 2.67 (0.82-8.64) | | 3.16 (0.33-29.75) | |
| ***GSTT1*** |  |  |  |  |  |  |  |  |
| Present | 1 (1.4) | 68 (98.6) | 18 (26.1) | 51 (73.9) | 24 (57.1) | 18 (42.9) | 55 (91.7) | 5 (8.3) |
| Null | 1 (6.7) | 14 (93.3) | 6 (40.0) | 9 (60.0) | 2 (28.6) | 5 (71.4) | 13 (100.0) | 0 (0.0) |
| *p*-value | 0.27 | | 0.28 | | 0.18 | | 1.00 | |
| OR (IC 95%) | 0.21 (0.01-3.49) | | 0.53 (0.16-1.70) | | 3.33 (0.58-19.18) | | NE | |
| ***GSTP1*** |  |  |  |  |  |  |  |  |
| IleIle | 2 (5.0) | 38 (95.0) | 12 (30.0) | 28 (70.0) | 11 (50.0) | 11 (50.0) | 31 (93.9) | 2 (6.1) |
| IleVal or ValVal | 0 (0.0) | 44 (100.0) | 12 (27.3) | 32 (72.7) | 15 (55.6) | 12 (44.4) | 37 (92.5) | 3 (7.5) |
| *p*-value | 1.00 | | 0.78 | | 0.70 | | 0.81 | |
| OR (IC 95%) | NE | | 1.14 (0.44-2.95) | | 0.80 (0.26-2.47) | | 1.26 (0.20-8.00) | |
| IleIle or IleVal | 2 (2.5) | 78 (97.5) | 23 (28.8) | 57 (71.3) | 24 (52.2) | 22 (47.8) | 65 (92.9) | 5 (7.1) |
| ValVal | 0 (0.0) | 4 (100.0) | 1 (25.0) | 3 (75.0) | 2 (66.7) | 1 (33.3) | 3 (100.0) | 0 (0.0) |
| *p*-value | 1.00 | | 0.87 | | 0.63 | | 1.00 | |
| OR (IC 95%) | NE | | 1.21 (0.12-12.25) | | 0.54 (0.05-6.44) | | NE | |

**Table S3.** *GSTM1*, *GSTT1* e *GSTP1* Ile105Val genotypes in head and neck squamous cell carcinoma patients stratified by hematological toxicity and response rate to chemoradiotherapy. (n) number of patients; (G) grade of toxicity; (CR) complete response; (PR) particle response; (SD) stable disease; (OR) odds ratio; (CI) confidence interval; (NE) not evaluated. *The total number of patients differed from the total (n = 90), because it was not possible to obtain consistent information about hematological data and response rate in some cases.
